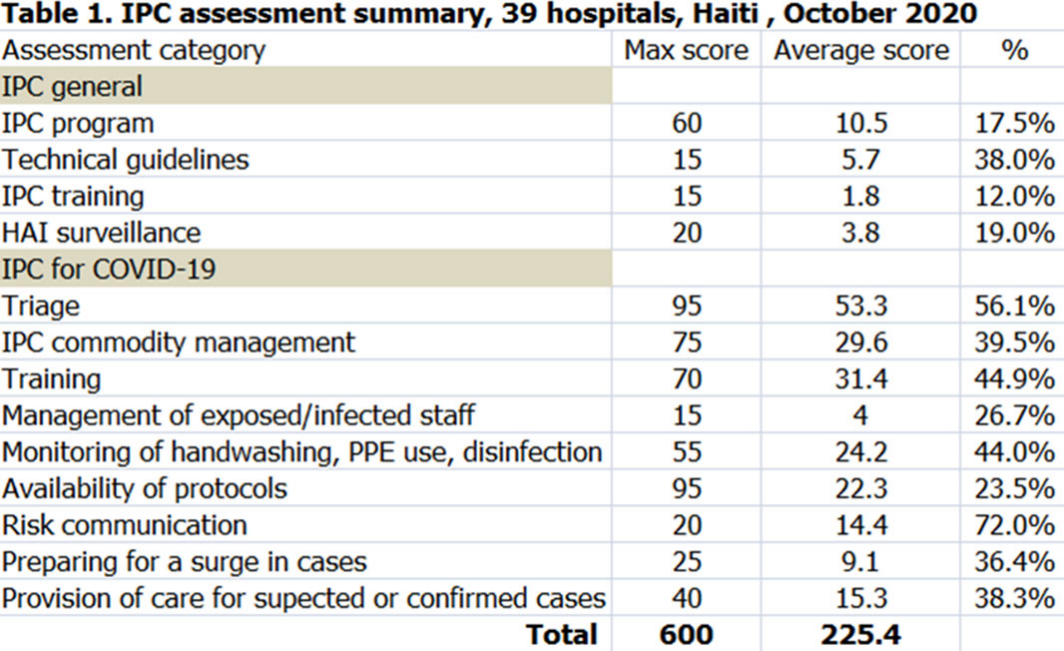# Assessment of COVID-19 Infection Prevention and Control Capabilities in 39 Haitian Hospitals

**DOI:** 10.1017/ash.2021.129

**Published:** 2021-07-29

**Authors:** Afeke Kambui, Mentor Lucien, Catherine Emilien, Francois Staco, Ymeline Pateau St Vil, Pierre Philippe Wilson Registe, Mackenley Brice, Dassaëve Brice, Nathan Zephirin, Sandra Benjamin, Martha Murdock

## Abstract

**Background:** Infection prevention and control (IPC) is key (1) to keeping health workers and patients safe from contracting infections during care, (2) to enabling continuity of essential health services, and (3) to pandemic preparedness and response. Frontline health workers are at 3-fold increased risk for COVID-19 (*Lancet* 2020) and account for 6% of COVID-19 hospitalizations (CDC 2020). With the support of the US Agency for International Development Bureau of Humanitarian Assistance (USAID/BHA) and collaboration of the Haitian Ministry of Health (MSPP), MSH’s Rapid Support to COVID-19 Response in Haiti project (RSCR Haiti) developed an instrument to assess select public hospitals and identify IPC gaps that informed COVID-19 response and system strengthening measures for increasing patient and provider safety. **Methods:** The IPC tool contains 13 IPC domains and 80 questions, for a total of 600 points. It was developed based on the World Health Organization IPC Assessment Framework for Health Facilities (2018) and US Centers for Disease Control Facility Readiness Assessment for COVID-19 (2020). In total, 39 health facilities chosen by the MSPP across all 10 departments of Haiti were evaluated in October 2020. Data were analyzed in Microsoft Excel by category, site, and IPC capabilities then classified as inadequate, basic, intermediate or advanced. **Results:** IPC capabilities scored as inadequate in 18% and basic in 67% of hospitals (Graph 1). No institution was advanced. Among health facilities, IPC programs existed in only 18%; IPC guidelines or procedures were present in 38%; staff were trained regularly in 12%; and healthcare-associated infection surveillance was performed in 19%. Systems for COVID-19 triage existed in 56%; 39% had IPC commodity management systems; 45% provided COVID-19 training; 26% practiced monitoring of staff and patients for COVID-19; 36% had protocols for an influx of COVID-19 cases; and 72% practiced risk communication (Table 1). **Conclusions:** No health facility was sufficiently equipped to implement adequate COVID-19 IPC measures, and all needed strengthening, even in the highest-scoring IPC areas. Through RSCR Haiti, MSH and MSPP were able to identify and address priorities in hospitals: establishing hospital IPC programs; training staff; monitoring health workers and patients; and implementing guidance, triage, and commodity-management systems. This study demonstrates that it is possible to do a quick yet thorough assessment to rapidly identify IPC needs and opportunities, using the results to rapidly build response capacity. Haiti’s experience of integrating locally contextualized global IPC tools to inform systemic COVID-19 response measures can benefit other experts globally.

**Funding:** United States Agency for International Development Bureau of Humanitarian Assistance (USAID/BHA)

**Disclosures:** None

**Figure 1.**
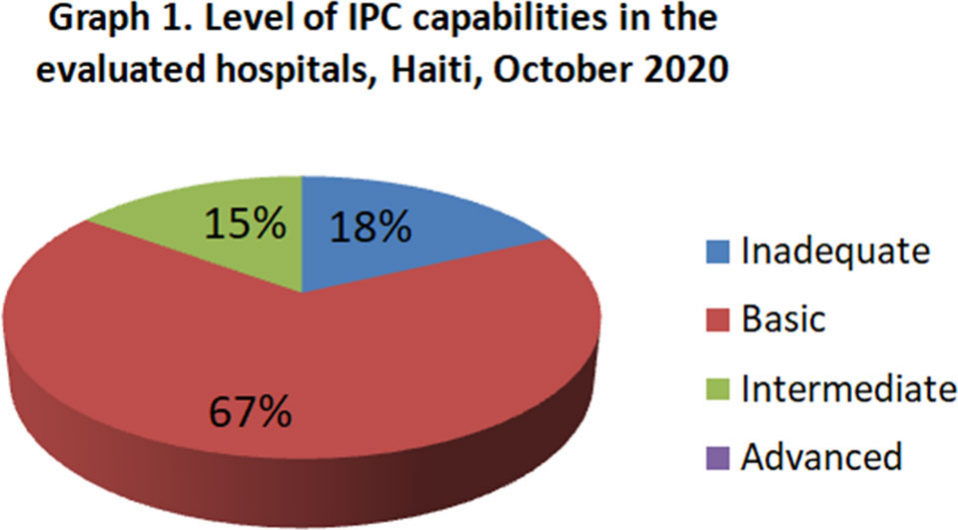

**Table 1.**